# Buprenorphine-Naloxone for Opioid Use Disorder: Reduction in Mortality and Increased Remission

**DOI:** 10.5811/westjem.18569

**Published:** 2024-09-06

**Authors:** Krishna K. Paul, Christian G. Frey, Stanley Troung, Laura vita Q. Paglicawan, Kathryn A. Cunningham, T. Preston Hill, Lauren G. Bothwell, Georgiy Golovko, Yeoshina Pillay, Dietrich Jehle

**Affiliations:** * Department of Emergency Medicine, University of Texas Medical Branch, Galveston, Texas; † Department of Pharmacology and Toxicology, University of Texas Medical Branch, Galveston, Texas; ‡ Department of Emergency Medicine, Michigan State University, East Lansing, Michigan

## Abstract

**Introduction:**

As fentanyl has become more readily available, opioid-related morbidity and mortality in the United States has increased dramatically. Preliminary studies suggest that high-affinity, partial mu-opioid receptor agonists such as the combination product buprenorphine-naloxone may reduce mortality from overdose and promote remission. With the escalating prevalence of opioid use disorder (OUD), it is essential to evaluate the effectiveness of opioid agonists like buprenorphine-naloxone. This study examines mortality and remission rates for OUD patients prescribed buprenorphine-naloxone to determine the efficacy of this treatment toward these outcomes.

**Methods:**

We carried out a retrospective analysis using the US Collaborative Network database in TriNetX, examining de-identified medical records from nearly 92 million patients across 56 healthcare organizations. The study spanned the years from January 1, 2017–May 13, 2022. Cohort 1 included OUD patients who began buprenorphine-naloxone treatment within one-year post-diagnosis, while Cohort 2, the control group, consisted of OUD patients who were not administered buprenorphine. The study measured mortality and remission rates within a year of the index event, incorporating propensity score matching for age, gender, and race/ethnicity.

**Results:**

Prior to propensity matching, we identified a total of 221,967 patients with OUD. Following exclusions, 61,656 patients treated with buprenorphine-naloxone showed 34% fewer deaths within one year of diagnosis compared to 159,061 patients who did not receive buprenorphine (2.6% vs 4.0%; relative risk [RR] 0.661; 95% confidence interval [CI] 0.627–0.698; *P* < 0.001). The remission rate was approximately 1.9 times higher in the buprenorphine-naloxone group compared to the control group (18.8% vs 10.1%; RR 1.862; 95% CI 1.812–1.914; *P* < 0.001). After propensity matching, the effect on mortality decreased but remained statistically significant (2.6% vs 3.0%; RR 0.868; 95% CI 0.813–0.927; *P* < 0.001) and the remission rate remained consistent (18.8% vs 10.4%; RR 1.812; 95% CI 1.750–1.876; *P* < 0.001). Number needed to treat for benefit was 249 for death and 12 for remission.

**Conclusion:**

Buprenorphine-naloxone was associated with significantly reduced mortality and increased remission rates for patients with opioid use disorder and should be used as a primary treatment. The recognition and implementation of treatment options like buprenorphine-naloxone is vital in alleviating the impact of OUD.

Population Health Research Capsule What do we already know about this issue?

*Opioid-related morbidity and mortality has increased in the US, along with fentanyl use. Studies show buprenorphine-naloxone to be an effective treatment for opioid use disorder (OUD).*

What was the research question?

*How are the mortality and remission rates of OUD patients affected by the prescription of buprenorphine-naloxone?*

What was the major finding of the study?

*Patients prescribed buprenorphine-naloxone had 34% fewer deaths (CI 0.63–0.70; *P* < 0.001) and 1.9 times more remission (CI 1.81–1.91; *P* < 0.001).*

How does this improve population health?

*As the incidence of OUD and the availability of fentanyl increases, healthcare interventions are essential. Buprenorphine-naloxone is an effective treatment option.*

## INTRODUCTION

### Background

The incidence and prevalence of opioid use disorder (OUD) is increasing both in the United States and globally. The recent proliferation of the illicit drug fentanyl has only intensified the potential for opioid abuse and raised the mortality rate.^1^ Opioid-related deaths have surged nearly four-fold since 1999, and mortality rates have continued their upward trajectory with the advent of synthetic opioids.^2^ ^–^ ^4^ Fentanyl has notably become the primary driver of drug-related overdoses, with an almost 7.5-fold increase in overdose-related deaths from 2015 to 2021.^2^ However, there is a range of US Food and Drug Administration-approved medications available for OUD that can reduce overdose-related mortality and promote remission.

Partial opioid agonist medications, such as buprenorphine, which can be found as a mono-product form or combined with naloxone (such as brand name Suboxone), have demonstrated efficacy in reducing the risk of overdose-related death compared to pure opioid antagonists like naltrexone.^5^ ^–^ ^8^ Buprenorphine is a partial opioid agonist that functions by binding to the mu-opioid receptor with particularly high affinity when the receptor is empty, thereby blocking the binding of other opioids with abuse potential while also alleviating withdrawal symptoms and cravings.^9^ However, when the receptor is occupied, buprenorphine dislodges the opioid from the receptor, thus precipitating withdrawal.

Buprenorphine-naloxone is distinctive in that when given as a combination, it can be used to mitigate possible inappropriate usage of buprenorphine alone. When administered sublingually, the naloxone component has little to no effect due to high first-pass hepatic metabolism. However, if buprenorphine-naloxone were to be used intravenously or intranasally, the naloxone can precipitate withdrawal as well as diminish any perceived euphoria.^9^ This unique characteristic of buprenorphine-naloxone greatly enhances its potential to reduce opioid-related overdoses. A study conducted over a 22-year period, published in 2020, revealed that the relative risk of overdose-related death was up to 3.2 times higher in the absence of opioid agonist therapy with buprenorphine.^8^ Moreover, prior research demonstrates potential in decreasing future illicit opioid use following initiation of treatment with buprenorphine-naloxone.^1^ A 2021 study found that buprenorphine-naloxone therapy was associated with significantly lower odds of fentanyl exposure over time compared to methadone or slow-release morphine treatment.^1^ This positions buprenorphine-naloxone as a uniquely effective combination of drugs used in the treatment of OUD.

### Importance

As the prevalence of OUD continues to rise, it is essential to identify a treatment that effectively reduces overdose-related mortality and increases remission rates.

### Goals of Investigation

In this study we used electronic health records (EHR) from the United States Collaborative Network in TriNetX to perform an analysis comparing patients prescribed buprenorphine-naloxone within one year of their OUD diagnosis with those who did not use opioid agonist therapy. The investigation examined mortality and remission rates between these two patient cohorts.

## METHODS

### Study Design

TriNetX is a global collaborative network consisting of de-identified patient EHR data from around the world. All cohort and outcome definitions were based on the International Classification of Diseases, 10^th^ Rev, Procedure Coding System, Clinical Modification (ICD-10-CM) entered into the health record systems. We identified medications prescribed using RxNorm codes (Table 1). RxNorm is a standardized nomenclature for clinical drugs and drug delivery devices in the US, developed and maintained by the National Library of Medicine. Using the US Collaborative Network of TriNetX, which contains approximately 92 million patients from 56 healthcare organizations (HCO) in the US, we established two cohorts.

**Table 1.** International Classification of Diseases and RxNorm codes for buprenorphine-naloxone and opioid use disorder.

Type	Name	Coding system	Code
Diagnosis	Opioid dependence, uncomplicated	ICD-10-CM	F11.20
Diagnosis	Opioid dependence, in remission	ICD-10-CM	F11.21
Diagnosis	Opioid abuse, in remission	ICD-10-CM	F11.11
Diagnosis	Other long-term (current) drug therapy	ICD-10-CM	Z79.899
Medication	Buprenorphine	RxNorm	1819
Medication	Naloxone	RxNorm	7242

*ICD-10-CM*, International Classification of Diseases; 10^th^ Rev, Clinical Modification; *OUD*, opioid use disorder.

### Cohort Definition

Cohort 1 included all patients diagnosed with uncomplicated opioid dependence (ICD-10-CM: F11.20) who were given a prescription of buprenorphine-naloxone (buprenorphine - RxNorm:1819 + naloxone -RxNorm:7242) within one year of any F11.20 diagnosis input into the health record system. Cohort 2 (control) consisted of patients diagnosed with uncomplicated opioid dependence with no current or prior prescription of buprenorphine. This was considered the starting event or “index event.” Individuals who had never been prescribed buprenorphine were selected as the control group, because defining the control group as those not having buprenorphine-naloxone listed in the TriNetX database could inadvertently have excluded patients who had been treated solely with naloxone following an overdose episode. For both cohorts, the time window was established between January 1, 2017–May 13, 2022. This time frame was chosen as buprenorphine-naloxone became widely accessible and prescribed from 2017 onward, and the end date of 2022 ensured there was at least one year between the date the patient was seen for OUD and one year of follow-up for outcomes.

### Outcomes

The two outcomes measured in these cohorts were mortality and remission. Remission was defined by the diagnosis of remission from opioid dependence (ICD-10-CM:F11.21), remission from opioid abuse (ICD-10-CM:F11.11), or the use of other long-term drug therapy (ICD-10-CM:Z79.899). The time window for these outcomes ranged from one day to one year following the index event, which was defined as the usage of buprenorphine-naloxone or non-usage of buprenorphine for OUD. We excluded from the study patients with remission prior to the index event. Mortality data within the TriNetX platform was obtained from EHR data and HCOs, in conjunction with the national death registries. There is potential for missed death events when a patient is treated at an HCO not affiliated with the TriNetX network and subsequently experiences a fatal outcome outside this network. However, this represents only a minor issue, as currently, 94% of HCOs within the TriNetX network are also linked to the US death registries. This percentage is steadily increasing as more HCOs continue to be linked with the registries.

### Secondary Analysis on Socioeconomic Status

We performed a secondary analysis to evaluate the impact of socioeconomic status on the prescription of buprenorphine-naloxone and outcomes in OUD patients. The presence of the ICD-10-codes Z56.0 (Unemployment, unspecified) or Z59 (Problems related to housing and economic circumstances) was used as a marker for prior history of lower socioeconomic status and was extracted from each cohort.

A post-hoc analysis was performed from June 2, 2004 to June 2, 2023, in which we evaluated OUD patients who were prescribed suboxone within one month of the OUD diagnosis and excluded those on methadone or naltrexone. The OUD definition was expanded to include additional ICD 09/10-codes associated with opioid abuse and opioid use as well (Supplementary Table 1). Propensity matching was slightly more robust, including additional covariates such as social determinants, disorders related to drugs of abuse, and nicotine (Supplementary Table 2).

### Statistical Analysis

A 1:1 propensity score match was produced via TriNetX, using logistic and linear regression. We used greedy nearest-neighbor matching with a tolerance of 0.1 and a difference between propensity scores less than or equal to 0.1.^10^ Propensity matching was performed for demographics including age at the index event, race, ethnicity, and gender, using the Balance Cohorts tool in TriNetX. All demographic data was self-reported by patients and recorded by HCOs to HL7 administrative standards. We used the Measure of Association Analysis tool in TriNetX to calculate risk ratio (RR), 95% confidence interval (CI), and *P*-values (P) for outcome comparisons through univariate analysis. We calculated the number needed to treat for benefit (NNTB) manually for each outcome. Patients who met the outcome prior to the visit were excluded from their respective cohorts for the outcome analysis to ensure that patients who had the outcome prior to the index event were excluded. The final data was obtained and analysis conducted on May 13, 2023. Statistical significance was determined at a two-sided alpha <0.05. Because we used de-identified data from TriNetX, this study was determined to be exempt by the University of Texas Medical Branch (UTMB) IRB. The UTMB IRB determined that this project did not involve intervention or interaction with human subjects, and the data was de-identified per the de-identification standard defined in Section §164.514(a) of the HIPAA Privacy Rule. This formal determination by a qualified expert refreshed on December 2020.^10^

## RESULTS

In this analysis, there were a total of 221,967 patients prior to propensity matching, with 62,041 patients in Cohort 1 and 159,926 patients in Cohort 2. Regarding the outcome of mortality, after exclusions, Cohort 1 consisted of 61,656 patients, and Cohort 2 comprised 159,061 patients. For the outcome of remission, there were a much larger number of patients excluded because of prior history of remission. Cohort 1 included 37,199 patients, and Cohort 2 contained 110,726 patients. After propensity matching, Cohort 1 maintained the same number of patients for both outcomes. When propensity matching was applied, Cohort 2 included 61,746 and 44,284 patients for the outcomes of death and remission, respectively, after exclusions. (Table 2).

**Table 2.** Demographics before and after propensity score matching with Cohort 1 buprenorphine-naloxone and Cohort 2 opioid use disorder controls.

	Before propensity score matching				After propensity score matching			
Demographics	Cohort 1 (n = 62,041)	Cohort 2 (n = 159,926)	*P*-value	Std. Diff	Cohort 1 (n = 62,041)	Cohort 2 (n = 62,041)	*P*-value	Std. Diff
Mean age at index ± SD	39.5 ± 12.3	45.8 ± 16.5	<0.001	0.43	39.5 ± 12.3	39.5 ± 12.4	=0.80	<0.01
Female	27,979 (45.1%)	78,641 (49.2%)	<0.001	0.08	27,979 (45.1%)	27,975 (45.1%)	=0.98	<0.01
Male	34,057 (54.9%)	81,271 (50.8%)	<0.001	0.08	34,057 (54.9%)	34,064 (54.9%)	=0.97	<0.01
White	46,370 (74.7%)	110,815 (69.3%)	<0.001	0.12	46,370 (74.7%)	46,536 (75.0%)	=0.28	<0.01
AI/AN	564 (0.91%)	807 (0.51%)	<0.001	0.05	564 (0.91%)	412 (0.66%)	<0.001	0.03
NHPI	42 (0.07%)	140 (0.09%)	=0.14	0.01	42 (0.07%)	15 (0.02%)	<0.001	0.02
Unknown ethnicity	10,945 (17.6%)	49,014 (30.6%)	<0.001	0.31	10,945 (17.6%)	10,922 (17.6%)	=0.86	<0.01
Not Hispanic or Latino	47,161 (76.0%)	101,782 (63.6%)	<0.001	0.27	47,161 (76.0%)	47,249 (76.2%)	=0.56	<0.01
Hispanic or Latino	3,935 (6.34%)	9,130 (5.71%)	<0.001	0.03	3,935 (6.34%)	3,870 (6.24%)	=0.45	<0.01
Black	6,999 (11.3%)	22,711 (14.2%)	<0.001	0.09	6,999 (11.3%)	6,993 (11.3%)	=0.96	<0.01
Asian	193 (0.31%)	810 (0.51%)	<0.001	0.03	193 (0.31%)	169 (0.27%)	=0.21	<0.01
Unknown race	7,873 (12.7%)	24,643 (15.4%)	<0.001	0.08	7,873 (12.7%)	7,916 (12.8%)	=0.71	<0.01

*OUD*, opioid use disorder; *AI/AN*, American Indian or Alaskan Native; *NHPI*, Native Hawaiian or other Pacific Islander.

**Table 3.** Outcomes before and after propensity score matching with Cohort 1 buprenorphine-naloxone and Cohort 2 opioid use disorder controls.

	Cohort 1	Cohort 2	RR (95% CI)	*P*-value
Mortality before PSM	1,628 (2.64%)	6,352 (3.99%)	0.66 (0.63–0.70)	P < 0.001
Mortality after PSM	1,628 (2.64%)	1,878 (3.04%)	0.87 (0.81–0.93)	P < 0.001
Remission before PSM	6,984 (18.78%)	11,163 (10.08%)	1.86 (1.81–1.91)	P < 0.001
Remission after PSM	6,984 (18.78%)	4,589 (10.36%)	1.81 (1.75–1.88)	P < 0.001

*OUD*, opioid use disorder; *PSM*, propensity score matching; *RR*, relative risk; *CI*, confidence interval.

After propensity matching, OUD patients experienced 13% less deaths (2.6% vs 3.0%, RR 0.87, 95% CI 0.81–0.93, *P* < 0.001) and 81% greater remissions rates (18.8% vs 10.4%, RR 1.81, 95% CI 1.75–1.88, *P* < 0.001) in the year following prescription of buprenorphine-naloxone when compared to those who were not prescribed buprenorphine. Before propensity matching, trends were similar; however, the effect of mortality was more pronounced (RR 0.66) (Table 3). The NNTB was 249 for death and 12 for remission within one year of index event.

The secondary analysis for the impact of socioeconomic status showed patients prescribed buprenorphine-naloxone were more likely to have a history of unemployment than those not prescribed buprenorphine (6.36% vs 2.54%, *P* < 0.001). Similarly, patients prescribed buprenorphine-naloxone were more likely to have had problems related to housing and economic circumstances than those not prescribed buprenorphine (13.93% vs 7.82%, *P* < 0.001). The post hoc analysis showed that after propensity matching for social determinants of health and other substance-related disorders, the relative risk of death in one year was 0.78 (95% CI 0.74–0.83; *P* < 0.001) and remission was 2.15 (95% CI 2.09–2.22; *P* < 0.001) for the patients prescribed buprenorphine-naloxone within one month of OUD diagnosis (Supplementary Table 3). Trends were similar before propensity matching. NNTB was 137 for death and 12 for remission.

## DISCUSSION

This multicenter, retrospective study has demonstrated that buprenorphine-naloxone use was assocaited with significantly lower mortality rates and higher remission rates in comparison to no treatment in patients with OUD. The utilization of the United States Collaborative Network in TriNetX provides the largest sample size in the current literature (221,967 before propensity matching) comparing buprenorphine-naloxone to no intervention. This large-dataset approach has mitigated potential confounding variables, including social determinants, that might have influenced the findings of previous research.

The findings of our study are consistent with other literature examining outcomes of medications for OUD. The Prescription Opioid Addiction Treatment Study trial demonstrated more successful outcomes (abstinence or near-abstinence from opioids) in prescription opioid users maintained on buprenorphine-naloxone than in those who were tapered off buprenorphine-naloxone.^11^ Only 7% of study participants who were tapered off buprenorphine-naloxone achieved successful outcomes compared to 49% who were maintained on buprenorphine-naloxone.^11^ This population of prescription opioid users is slightly different from our population of patients with OUD. Other studies have compared the use of buprenorphine-naloxone with other methods, such as extended-release naltrexone, or methadone, and have found either intervention to be equally safe and effective.^12^ ^–^ ^14^ Patients were even found to have greater short-term success in treatment with buprenorphine-naloxone compared to clonidine in a small, multicenter, randomized trial.^15^

Another study considered no treatment, inpatient detoxification, behavioral health, buprenorphine or methadone, naltrexone, and non-intensive behavioral health interventions in individuals with OUD. Only treatment with buprenorphine or methadone demonstrated a significantly reduced risk of overdose and opioid-related acute care at 3- and 12-month follow-ups.^16^ Our study further strengthens the argument for medication use in treating OUD and demonstrates the effectiveness of buprenorphine-naloxone.

The findings of this study carry significant implications for the acute management of OUD. With the global rise in OUD, exacerbated by the increased availability of fentanyl, mitigating mortality rates for individuals with OUD remains a major public health challenge.^17^ These individuals may require medical interventions for the treatment of complications of OUD such as autonomic instability, hypoxia, endocarditis, ischemia, or cardiac arrest.^18^ Proactive pharmacologic management is a key component in preventing the life-threatening consequences of OUD.

## LIMITATIONS

It is important to acknowledge the inherent limitations of a retrospective study, especially in that causation cannot be established. This study included a sample of 221,967 patients from 56 HCOs before propensity matching, which is more extensive than previous studies, thereby providing increased statistical power. The large sample size and use of propensity matching help mitigate some of these limitations. Propensity matching was performed for covariates including age, gender, race, and ethnicity, but there may be other unaccounted variables that could have influenced the mortality rates or remission in this group. Due to de-identification and privacy policies inherent to the TriNetX database, granular and non-codable data such as social determinants are limited, while geographic identifiers such as ZIP codes are unobtainable. Moreover, this study did not take into account comorbid medical conditions or psychiatric illnesses, which might also have impacted the outcomes.

The database records prescriptions in the health records but does not report the dosages of buprenorphine-naloxone, exact timing of this adjunct therapy, or patient adherence to regimen. This omission is significant because different medication dosages and compliance may affect the efficacy of buprenorphine-naloxone. For the control group, alternative methods for managing OUD may have been present but were not considered in the analysis. Additionally, without knowing how long a patient was drug free, we could not rule out potential relapses as a cause for the index visit in the excluded group. Furthermore, the parameter of the data collected restricts the generalization of the study for international populations and long-term results beyond a year.

Finally, the study evaluated the parameters of mortality rate and remission, but other measures weren’t included. We did not consider quality of life measures, which could provide a more comprehensive understanding of the effects of buprenorphine-naloxone on lifestyle in those with OUD. Future research should focus on other life outcome measures, social determinants, and the long-term impacts of buprenorphine-naloxone on the individual.

## CONCLUSION

This multicenter retrospective analysis shows that buprenorphine-naloxone use is associated with significantly improved mortality rates compared to no intervention in patients with opioid use disorder. Furthermore, the study highlights an association with higher remission rates in this population. While these findings, along with previous studies, suggest that buprenorphine-naloxone is an effective treatment option for OUD, further prospective studies comparing to other treatment modalities should be considered to confirm efficacy.

## Supplementary Information




