# Supplementary material for: Buprenorphine-Naloxone for Opioid Use Disorder: Reduction in Mortality and Increased Remission
Source: West J Emerg Med. 2024 Sep 6;25(6):869–74. doi: 10.5811/westjem.18569 (PMC11610725; doi:10.5811/westjem.18569)
Supplement: Supplementary file 1 [file wjem-25-869-s001.docx]

Supplementary Appendix – Post Hoc Analysis

| **Table 1: Post-Hoc ICD-10-CM and RxNorm Codes for Buprenorphine-Naloxone and OUD** | | | |
| --- | --- | --- | --- |
| **Type** | **Name** | **Coding System** | **Code** |
| Diagnosis | Opioid abuse, uncomplicated | ICD-10-CM | F11.10 |
| Diagnosis | Opioid abuse with intoxication | ICD-10-CM | F11.12 |
| Diagnosis | Opioid abuse with withdrawal | ICD-10-CM | F11.13 |
| Diagnosis | Opioid abuse with opioid-induced mood disorder | ICD-10-CM | F11.14 |
| Diagnosis | Opioid abuse with opioid-induced psychotic disorder | ICD-10-CM | F11.15 |
| Diagnosis | Opioid abuse with other opioid-induced disorder | ICD-10-CM | F11.18 |
| Diagnosis | Opioid abuse with unspecified opioid-induced disorder | ICD-10-CM | F11.19 |
| Diagnosis | Opioid dependence, uncomplicated | ICD-10-CM | F11.20 |
| Diagnosis | Opioid dependence with intoxication | ICD-10-CM | F11.22 |
| Diagnosis | Opioid dependence with withdrawal | ICD-10-CM | F11.23 |
| Diagnosis | Opioid dependence with opioid-induced mood disorder | ICD-10-CM | F11.24 |
| Diagnosis | Opioid dependence with opioid-induced psychotic disorder | ICD-10-CM | F11.25 |
| Diagnosis | Opioid dependence with other opioid-induced disorder | ICD-10-CM | F11.28 |
| Diagnosis | Opioid dependence with unspecified opioid-induced disorder | ICD-10-CM | F11.29 |
| Diagnosis | Opioid type dependence | ICD-09-CM | 304.0 |
| Diagnosis | Opioid use, unspecified, uncomplicated | ICD-10-CM | F11.90 |
| Diagnosis | Opioid use, unspecified with intoxication | ICD-10-CM | F11.92 |
| Diagnosis | Opioid use, unspecified with withdrawal | ICD-10-CM | F11.93 |
| Diagnosis | Opioid use, unspecified with opioid-induced mood disorder | ICD-10-CM | F11.94 |
| Diagnosis | Opioid use, unspecified with opioid-induced psychotic disorder | ICD-10-CM | F11.95 |
| Diagnosis | Opioid use, unspecified with other specified opioid-induced disorder | ICD-10-CM | F11.98 |
| Diagnosis | Opioid use, unspecified with unspecified opioid-induced disorder | ICD-10-CM | F11.99 |
| Diagnosis | Opioid dependence, in remission | ICD-10-CM | F11.21 |
| Diagnosis | Opioid abuse, in remission | ICD-10-CM | F11.11 |
| Diagnosis | Other long term (current) drug therapy | ICD-10-CM | Z79.899 |
| Medication | Methadone | RxNorm | 6813 |
| Medication | Naltrexone | RxNorm | 7243 |
| Medication | Buprenorphine | RxNorm | 1819 |
| Medication | Naloxone | RxNorm | 7242 |

| **Table 2: Post-Hoc Demographics Before and After Propensity Score Matching with Cohort 1 Buprenorphine-Naloxone and Cohort 2 OUD Controls** | | | | | | | | | |
| --- | --- | --- | --- | --- | --- | --- | --- | --- | --- |
|  | | **Before Propensity Matching** | | | | **After Propensity Matching** | | | |
| **Cohort** | **Demographics** | **Outcome** | **%** | **p-value** | **Std. Diff** | **Outcome** | **%** | **p-value** | **Std. Diff** |
| 1*  2** | Age at Index (Mean±SD) | 38.8±12.3 44.6±16.9 | 100% 100% | <0.001 | 0.389 | 38.9±12.3 38.9±12.5 | 100% 100% | 0.940 | <0.001 |
| 1  2 | Female | 39,081 227,726 | 41.3% 44.1% | <0.001 | 0.057 | 38,589 38,019 | 41.4% 40.8% | 0.007 | 0.012 |
| 1  2 | Black or African American | 9,472 75,342 | 10.0% 14.6% | <0.001 | 0.140 | 9,442 10,334 | 10.1% 11.1% | <0.001 | 0.031 |
| 1  2 | Male | 52,160 272,839 | 55.1% 52.8% | <0.001 | 0.045 | 51,278 51,944 | 55.0% 55.7% | 0.002 | 0.014 |
| 1  2 | White | 72,003 349,886 | 76.0% 67.7% | <0.001 | 0.185 | 70,734 69,961 | 75.8% 75.0% | <0.001 | 0.019 |
| 1  2 | American Indian or Alaska Native | 641 1,934 | 0.7% 0.4% | <0.001 | 0.042 | 623 589 | 0.7% 0.6% | 0.327 | 0.005 |
| 1  2 | Unknown Race | 8,905 67,795 | 9.4% 13.1% | <0.001 | 0.118 | 8,824 8,604 | 9.5% 9.2% | 0.080 | 0.008 |
| 1  2 | Native Hawaiian or Other Pacific Islander | 190 1,858 | 0.2% 0.4% | <0.001 | 0.030 | 190 252 | 0.2% 0.3% | 0.003 | 0.014 |
| 1  2 | Unknown Gender | 3,498 16,133 | 3.7% 3.1% | <0.001 | 0.031 | 3,430 3,334 | 3.7% 3.6% | 0.234 | 0.006 |
| 1  2 | Not Hispanic or Latino | 74,565 348,771 | 78.7% 67.5% | <0.001 | 0.255 | 73,245 73,282 | 78.5% 78.5% | 0.835 | 0.001 |
| 1  2 | Hispanic or Latino | 5,904 36,134 | 6.2% 7.0% | <0.001 | 0.031 | 5,864 5,791 | 6.3% 6.2% | 0.485 | 0.003 |
| 1  2 | Other Race | 3,144 15,440 | 3.3% 3.0% | <0.001 | 0.019 | 3,104 3,146 | 3.3% 3.4% | 0.589 | 0.003 |
| 1  2 | Asian | 384 4,443 | 0.4% 0.9% | <0.001 | 0.057 | 380 411 | 0.4% 0.4% | 0.269 | 0.005 |
|  | **Social Determinants** |  |  |  |  |  |  |  |  |
| 1  2 | Homelessness | 4,590 8,583 | 4.8% 1.7% | <0.001 | 0.180 | 4,196 4,282 | 4.5% 4.6% | 0.339 | 0.004 |
| 1  2 | Problems related to employment and unemployment | 2,242 3,059 | 2.4% 0.6% | <0.001 | 0.147 | 1,967 1,754 | 2.1% 1.9% | <0.001 | 0.016 |
|  | **Substance related disorders** |  |  |  |  |  |  |  |  |
| 1  2 | Nicotine dependence | 43,919 125,859 | 46.4% 24.4% | <0.001 | 0.473 | 42,523 42,809 | 45.6% 45.9% | 0.184 | 0.006 |
| 1  2 | Alcohol related disorders | 16,772 45,714 | 17.7% 8.8% | <0.001 | 0.263 | 15,984 17,138 | 17.1% 18.4% | <0.001 | 0.032 |
| 1  2 | Cannabis related disorders | 13,355 28,461 | 14.1% 5.5% | <0.001 | 0.292 | 12,415 12,977 | 13.3% 13.9% | <0.001 | 0.018 |
| 1  2 | Cocaine related disorders | 12,439 20,012 | 13.1% 3.9% | <0.001 | 0.337 | 11,262 11,517 | 12.1% 12.3% | 0.071 | 0.008 |
| 1  2 | Other stimulant related disorders | 10,739 14,312 | 11.3% 2.8% | <0.001 | 0.339 | 9,582 9,514 | 10.3% 10.2% | 0.603 | 0.002 |
| OUD, Opioid Use Disorder; *Cohort 1, Patients prescribed buprenorphine-naloxone; **Cohort 2, Patients not prescribed buprenorphine | | | | | | | | | |

| **Table 3: Post-Hox Outcomes before and after Propensity Score Matching with Cohort 1 Buprenorphine-Naloxone and Cohort 2 OUD Controls** | | | | |  |
| --- | --- | --- | --- | --- | --- |
|  | **Cohort 1** | **Cohort 2** | **RR (95% CI)** | **P-Value** | |
| **Mortality before PSM** | 2,503 (2.66%) | 20,447 (3.98%) | 0.67 (0.64-0.70) | P<0.001 | |
| **Mortality after PSM** | 2,445 (2.64%) | 3,122 (3.37%) | 0.78 (0.74-0.83) | P<0.001 | |
| **Remission before PSM** | 11,021 (16.61%) | 29,015 (7.17%) | 2.32 (2.27-2.36) | P<0.001 | |
| **Remission after PSM** | 10,856 (16.55%) | 5,397 (7.69%) | 2.15 (2.09-2.22) | P<0.001 | |
| PSM, Propensity Score Matching; RR, Relative Risk; CI, Confidence Interval; P, P-Value | | | | |  |
